# Clinical effectiveness of patellar resurfacing, no resurfacing and selective resurfacing in primary total knee replacement: systematic review and meta-analysis of interventional and observational evidence

**DOI:** 10.1186/s12891-022-05877-7

**Published:** 2022-10-22

**Authors:** Michal Grela, Matthew Barrett, Setor K. Kunutsor, Ashley W. Blom, Michael R. Whitehouse, Gulraj S. Matharu

**Affiliations:** 1 Musculoskeletal Research Unit, Bristol Medical School, University of Bristol, Level 1 Learning and Research Building, Southmead Hospital, Westbury-On-Trym, Bristol, BS10 5NB UK; 2 National Institute for Health Research Bristol Biomedical Research Centre, Bristol, UK

**Keywords:** Primary total knee replacement, Patellar resurfacing, Selective resurfacing, No resurfacing, Outcomes, Meta-analysis

## Abstract

**Background:**

Patellar resurfacing is optional during total knee replacement (TKR). Some surgeons always resurface the patella, some never resurface, and others selectively resurface. Which resurfacing strategy provides optimal outcomes is unclear. We assessed the effectiveness of patellar resurfacing, no resurfacing, and selective resurfacing in primary TKR.

**Methods:**

A systematic review and meta-analysis was performed. MEDLINE, Embase, Web of Science, The Cochrane Library, and bibliographies were searched to November 2021 for randomised-control trials (RCTs) comparing outcomes for two or more resurfacing strategies (resurfacing, no resurfacing, or selective resurfacing) in primary TKR. Observational studies were included if limited or no RCTs existed for resurfacing comparisons. Outcomes assessed were patient reported outcome measures (PROMs), complications, and further surgery. Study-specific relative risks [RR] were aggregated using random-effects models. Quality of the evidence was assessed using GRADE.

**Results:**

We identified 33 RCTs involving 5,540 TKRs (2,727 = resurfacing, 2,772 = no resurfacing, 41 = selective resurfacing). One trial reported on selective resurfacing. Patellar resurfacing reduced anterior knee pain compared with no resurfacing (RR = 0.65 (95% CI = 0.44–0.96)); there were no significant differences in PROMs. Resurfacing reduced the risk of revision surgery (RR = 0.63, CI = 0.42–0.94) and other complications (RR = 0.54, CI = 0.39–0.74) compared with no resurfacing. Quality of evidence ranged from high to very low. Limited observational evidence (5 studies, TKRs = 215,419) suggested selective resurfacing increased the revision risk (RR = 1.14, CI = 1.05–1.22) compared with resurfacing. Compared with no resurfacing, selective resurfacing had a higher risk of pain (RR = 1.25, CI = 1.04–1.50) and lower risk of revision (RR = 0.92, CI = 0.85–0.99).

**Conclusions:**

Level 1 evidence supports TKR with patellar resurfacing over no resurfacing. Resurfacing has a reduced risk of anterior knee pain, revision surgery, and complications, despite PROMs being comparable. High-quality RCTs involving selective resurfacing, the most common strategy in the UK and other countries, are needed given the limited observational data suggests selective resurfacing may not be effective over other strategies.

**Supplementary Information:**

The online version contains supplementary material available at 10.1186/s12891-022-05877-7.

## Introduction

Total knee replacement (TKR) is clinically and cost-effective for treating patients with painful arthritis [1, 2]. In the UK over 100,000 primary TKRs are performed annually [3, 4], with numbers rising [5]. Despite good TKR implant longevity [1–3], up to 34% of patients experience persistent pain [6], which can leave patients dissatisfied, with reduced mobility, and needing long-term analgesia [7]. Priority setting partnerships with patients and healthcare workers highlight chronic pain after TKR as an important area for future research [8, 9]. The reasons why many patients have pain after TKR remain unclear [10, 11], however it may relate to interventions performed during surgery.

When performing TKR, surgeons can retain the native patella, or resurface the patella (patellar resurfacing) using a polyethylene implant. Patellar resurfacing may be performed for a variety of indications including patient age, weight, patellar anatomy, the condition of the patella articular cartilage, presence of inflammatory arthritis, radiographic findings, and preoperative anterior knee pain [12, 13]. The National Joint Registry (NJR) recorded patellar resurfacing in 38% of 1,100,000 primary TKRs [3]. However rates vary substantially worldwide (USA 82% vs. Sweden 2%) [14].

Some surgeons always resurface the patella whilst others never do. Proponents of patellar resurfacing claim that if not resurfaced, 25% of patients develop chronic anterior knee pain with poor outcomes and dissatisfaction [15]. This adversely affects patient reported outcome measures (PROMs) and can lead to further surgery (secondary patellar resurfacing) in 7% [15]. Two-thirds of patients experience poor satisfaction after secondary patellar resurfacing [16, 17]. Opponents of patellar resurfacing propose that resurfacing is an unnecessary additional procedure given similar PROMs between those resurfaced and not [1]. Resurfacing also extends surgical time and increases the risk of intraoperative complications (e.g. patella fracture, tendon injury) [15].

The National Institute for Health and Care Excellence (NICE) guidance currently recommends always performing patellar resurfacing rather than not resurfacing [18]. However a third option exists (selective patellar resurfacing), where the surgeon decides case-by-case whether or not to resurface the patella based on their experience and intraoperative findings. Selective resurfacing could be a more effective strategy than always resurfacing, as it potentially preserves benefits from both approaches. Selective resurfacing may improve outcomes by only resurfacing patients whom surgeons judge are at higher risk of future pain if they were not resurfaced. Conversely by not resurfacing patients where the surgeon thinks resurfacing is not needed, or where there may be a high risk of complications, there are potential cost-savings from decreased theatre time and implants.

Limited evidence exists for selective patellar resurfacing with only one RCT published [19]. NICE were therefore unable to make recommendations about selective resurfacing; however NICE have recently recommended future RCTs comparing selective resurfacing with always resurfacing to define the role of selective resurfacing in TKR [18]. This is concerning, as a recent survey of 309 UK surgeons demonstrated the most common practice is selective patellar resurfacing (39%), followed by always resurfacing (37%), and no resurfacing (24%) [12]. Furthermore most selective patellar resurfacing surgeons (71%) resurface in less than 50% of TKRs [12], which is contrary to NICE recommendations [18]. Many countries, including Australia and New Zealand, also employ selective resurfacing in up to two-thirds of cases, for which there may be little supportive evidence [14, 20, 21]. However since NICE issued their guidance, 5 more RCTs have been published, and NICE did not consider observational evidence for selective resurfacing which is important given only 1 RCT exists involving selective resurfacing.

We performed a systematic review to determine the clinical effectiveness and complication risks of patellar resurfacing, no resurfacing, and selective resurfacing in primary TKR patients.

## Methods

### Data sources and search strategy

This review was registered with the prospective register of systematic reviews, PROSPERO (CRD42020182670) and conducted using PRISMA and MOOSE guidelines [22, 23]. (Supplementary Materials 1–2) We performed electronic searches of MEDLINE, Embase, Web of Science and Cochrane Library databases from inception to 06 November 2021 to identify studies comparing at least two of the three possible patellar resurfacing options in primary TKR patients; resurfacing, no resurfacing, or selective resurfacing. The computer-based searches combined free and MeSH search terms and key words related to the population (e.g., “total knee replacement”), intervention (e.g., “resurfacing”) and outcomes (e.g., “revision rate”). There were no language restrictions. Full details of the search strategy are reported (Supplementary Material 3).

Titles and abstracts of retrieved studies were initially screened to assess their suitability for inclusion by two independent reviewers (MJG and MCB). Full text evaluations of potentially relevant articles meeting the selection criteria were performed by the same two independent reviewers. Any disagreements regarding eligibility of a study were discussed, and if needed consensus was reached with a third author (SKK). Reference lists of identified studies and relevant review articles were scanned manually and the “Cited Reference Search” function in Web of Science was used to check for additional eligible studies.

### Study eligibility criteria

Studies were included in our analyses if they were RCTs that compared any two or more of the following patellar resurfacing strategies (patellar resurfacing, no resurfacing, or selective resurfacing) in adult patients (18 years and above) undergoing primary TKR with at least one outcome of interest reported. If limited or no RCTs were available for a comparison, we included observational studies (prospective or retrospective). This was decided *a priori* as it was suspected limited RCTs would be available for the selective resurfacing arm given the evidence supporting national recommendations [18].

Outcomes evaluated were postoperative PROMs (e.g. Western Ontario and McMaster Universities Osteoarthritis Index (WOMAC), Knee Society Score (KSS), range of movement (ROM), Knee Injury and Osteoarthritis Outcome Score (KOOS), Hospital for Special Surgery (HSS), Oxford Knee Score, Feller’s Patellar Score) and rates of postoperative complications, revision surgery and any reoperation. We excluded the following studies: (i) those restricted to selected patients such as those with prevalent conditions like diabetes or selected populations with no comparison or control groups; (ii) observational studies investigating patellar resurfacing vs. no resurfacing without a selective resurfacing arm; and (iii) studies of any surgery other than primary TKR (such as revision surgery or unicompartmental knee replacement). No limits were placed on study follow-up duration.

### Data extraction, risk of bias and methodological quality assessment

One author (MCB) initially extracted data from eligible studies using a standardised predesigned data collection form. Two reviewers (MJG and SKK) independently checked these data with those in the original articles. We extracted data on study characteristics, sample size, preoperative and postoperative PROMs, and counts of outcomes of interest for the intervention and comparator(s) where relevant. When further information was required from a study, we attempted to contact the corresponding authors. When multiple publications involving the same cohort existed, we used the most complete study with the longest follow-up and/or analysis covering the largest number of participants. The Cochrane Collaboration’s risk of bias tool was used to assess the risk of bias of included RCTs [24]. This tool evaluates seven possible sources of bias, which are random sequence generation, allocation concealment, blinding of participants and personnel, blinding of outcome assessment, incomplete outcome data, selective reporting and other bias. For each individual component, studies were classified into low, unclear and high risk of bias. The methodological quality of each observational study was assessed using the nine-star Newcastle–Ottawa Scale (NOS) [25], which uses three pre-defined domains including: (i) selection of participants; (ii) comparability; and (iii) ascertainment of outcomes of interest. To grade the quality of evidence across outcomes, we used the Grading of Recommendations Assessment, Development and Evaluation (GRADE) tool, a widely adopted reproducible and transparent framework for grading certainty in evidence [26]. GRADE considers the following criteria: study limitations, inconsistency of effect, imprecision, indirectness, and publication bias, and has four levels of evidence: very low, low, moderate, and high.

### Statistical analyses

Summary measures were presented as relative risks [RR] with 95% confidence intervals (CIs) for binary outcomes, and mean differences (95% CIs) for continuous outcomes. RRs were calculated from the extracted raw counts for the intervention and comparator. For continuous data, if the mean or standard deviation (SD) was not reported, we estimated the mean and variance from the reported median, range and sample size using methods proposed by Hozo and colleagues [27]. Given the heterogeneous follow-up periods reported by included studies, risk estimates for the longest follow-up of each study were used for the outcomes. The inverse variance weighted method was used to combine summary measures using random-effects models. Parallel analyses utilised fixed effects models. Statistical heterogeneity across studies was quantified using the Cochrane *χ*^*2*^ statistic and the *I*^*2*^statistic. Pre-specified study-level characteristics such as geographical location, study year, mean age at baseline, mean follow-up duration and sample size were explored as sources of heterogeneity, using stratified analysis and meta-regression [28]. STATA release MP 16 (StataCorp LP, College Station, TX, USA) was used for all statistical analyses.

## Results

### Study identification and selection

**Fig. 1:**
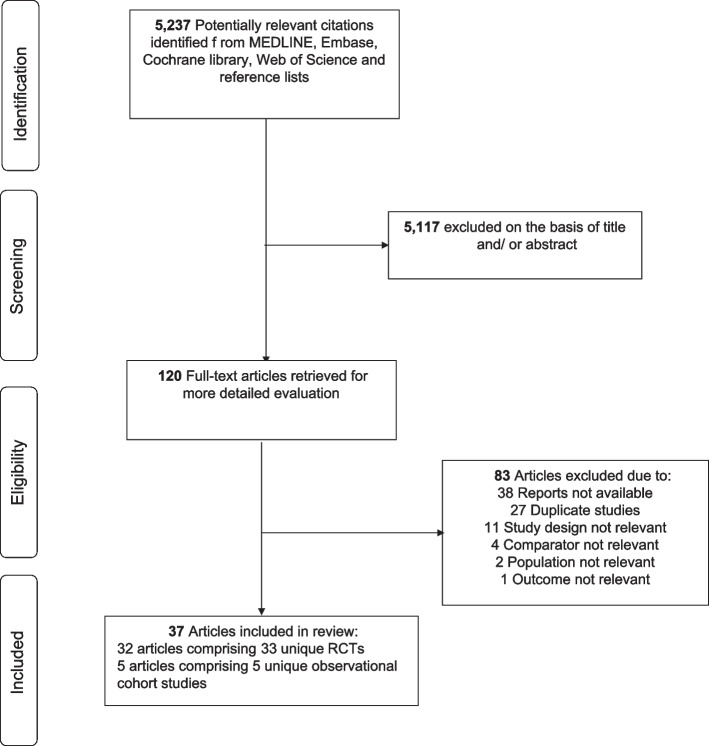
PRISMA flow diagram. RCT, randomised controlled trials

A total of 5,237 potential citations were identified from the initial search. Of these, 120 potential articles were selected for full text evaluation after screening the titles and abstracts. Following detailed evaluation of full texts, 83 citations were excluded. The remaining 37 articles comprising of 38 unique studies were eligible (Fig. 1; Supplementary Material 4). In total, there were 33 individual RCTs (based on 32 articles) that compared patellar resurfacing vs. no patellar resurfacing [1, 19, 29–58]. One trial had three arms (resurfacing vs. no resurfacing vs. selective resurfacing) [19]. We identified 5 unique observational studies that compared selective resurfacing with no resurfacing or patellar resurfacing [20, 21, 59–61].

### Study characteristics, risk of bias and methodological quality

The included studies were published from 1995 to 2021, with baseline characteristics of the individual RCTs summarised in Table 1. The 33 RCTs involved 5,540 primary TKRs (2,727 = resurfacing, 2,772 = no resurfacing, 41 = selective resurfacing). Overall, 14 studies [1, 19, 29–39] were conducted in Europe (Finland, France, Germany, Italy, the Netherlands, Norway, Serbia, Sweden and United Kingdom), 10 in Asia (China, India, Iran, Japan, South Korea and Thailand) [40–49], 5 in North America (Canada and USA) [50–54] and 4 in Australasia (Australia) [55–58] The mean baseline age of participants in the included studies ranged from 56.1 to 74.1 years with a weighted mean of 69.4 years. The mean follow-up periods in the trials ranged from 0.5 to 10 years with a weighted mean of 6.3 years. Using the Cochrane Risk of Bias tool, 11 trials demonstrated a low risk of bias in all domains [30, 34, 39, 42, 48, 51–54, 57, 58]. Five trials demonstrated a high risk of bias in one or more domains [1, 31, 38, 40, 45]. Except for 6 trials [31, 32, 35, 41, 47, 50], all trials demonstrated a low risk of bias in random sequence generation (Supplementary Material 5). Baseline characteristics of the observational studies are summarised in Supplementary Material 6. The 5 observational studies [20, 21, 59–61] included 4 retrospective cohort designs [20, 21, 59, 61] and 1 prospective cohort design [60], comprising a total of 215,419 primary TKRs. Studies were conducted in Australia, New Zealand, Korea, United Kingdom and USA. The mean baseline age of participants ranged from 64.3 to 68.7 years with mean follow-up periods ranging from 4.5 to 10.0 years. The NOS score ranged from 7 to 9 (Supplementary Material 7).

**Table 1:** Baseline characteristics of randomised controlled trials

**Author, year of publication**	**Location**	**Baseline year of study**	**Population**	**Mean/median age (years)**	**% Males**	**Mean follow-up duration (years)**	**Implant design**	**Interventions evaluated**	**No. of participants/** **joint replacements**
Partio, 1995 [38]	Finland	1991–1992	Osteoarthritis/Rheumatoid arthritis	67.5	22.0	3.0	Johnson & Johnson Press-fit condylar implant	Resurfacing/ No resurfacing	95
Feller, 1996 [56]	Australia	1990–1991	Osteoarthritis (not otherwise specified)	70.8	55.3	3.0	Howmedica PCA Modular prosthesis	Resurfacing/ No resurfacing	38
Kajino, 1997 [45]	Japan	NR	Rheumatoid arthritis	56.1	7.7	6.6	Yoshino-Shoji total knee prosthesis; Biomet, Warsaw, Indiana	Resurfacing/ No resurfacing	52
Schroeder-Boersch, 1998 [35]	Germany	NR	Primary osteoarthritis	72.6	30.0	2.0	Howmedica Duracon	Resurfacing/ No resurfacing	40
Newman, 2000 [19]	United Kingdom	1992–1997	Primary osteoarthritis	71.9	32.8	5.0	Howmedica PCA Modular prosthesis	Resurfacing/ No resurfacing/ Selective resurfacing	105
Wood, 2002 [58]	Australia	1992–1996	Non-inflammatory arthritis	73.7	52.7	4.0	Zimmer Miller-Galante II	Resurfacing/ No resurfacing	201
Mayman, 2003 [50]	Canada	1991	Osteoarthritis	70.0	42.0	10.0	Anatomic Medullary Knee, DePuy, Warsaw, IN	Resurfacing/ No resurfacing	100
Waters, 2003 [37]	United Kingdom	1992-	Osteoarthritis/inflammatory arthritis	69.1	40.2	5.3	Johnson & Johnson Press-fit condylar implant	Resurfacing/ No resurfacing	390
Burnett, 2004 [13, 51]	Canada	1991-	Osteoarthritis (not otherwise specified)	70	43.3	10.0	DePuy Anatomic Medullary Knee	Resurfacing/ No resurfacing	83
Gildone, 2005 [32]	Italy	2002–2004	Primary osteoarthritis	74.1	30.4	2.1	Zimmer Nexgen	Resurfacing/ No resurfacing	56
Campbell, 2006 [55]	Australia	1991–1993	Primary osteoarthritis	72.1	28.0	10.0	Zimmer Miller-Galante II	Resurfacing/ No resurfacing	100
Myles, 2006 [34]	United Kingdom	NR	Non-inflammatory arthritis	70	52.0	1.8	DePuy LCS rotating platform	Resurfacing/ No resurfacing	50
Smith, 2008 [57]	Australia	1998–2002	Primary osteoarthritis	71.5	50.4	4.0	Smith & Nephew PROFIX	Resurfacing/ No resurfacing	164
Burnett, 2009 [52]	USA	1992–1993	Degenerative osteoarthrosis	66.2	79.1	10.0	Zimmer Miller-Galante II	Resurfacing/ No resurfacing	86
Beaupre, 2012 [53]	Canada	1996–1999	Non-inflammatory arthritis	63.6	31.6	10.0	Profix™ Total Knee System	Resurfacing/ No resurfacing	38
Liu, 2012 [46]	China	2000–2002	Osteoarthritis	67.7	62.9	7.0	Press Fit Condylar, DePuy, Warsaw, IN	Resurfacing/ No resurfacing	144
Ferguson, 2014 [33] (Fixed bearing)	United Kingdom	NR	Osteoarthritis (not otherwise specified)	69.8	47.0	2.0	PFC Sigma© Posterior Stabilised, DePuy, Warsaw, IN	Resurfacing/ No resurfacing	176
Ferguson, 2014 [33] (Mobile Bearing)	United Kingdom	NR	Osteoarthritis (not otherwise specified)	70.2	47.0	2.0	PFC Sigma© Posterior Stabilised, DePuy, Warsaw, IN	Resurfacing/ No resurfacing	176
Murray, 2014 [1]	United Kingdom	1999–2003	Osteoarthritis/Rheumatoid arthritis	70	44.3	10.0	NR	Resurfacing/ No resurfacing	1715
Roberts, 2015 [54]	USA	1996–2001	Primary osteoarthritis	70.7	48.6	7.8	DePuy Sigma CR	Resurfacing/ No resurfacing	114
Aunan, 2016 [30]	Norway	2007–2011	Primary osteoarthritis	69.5	43.4	3.0	Zimmer Nexgen	Resurfacing/ No resurfacing	129
Ali, 2016 [29]	Sweden	2008–2009	Primary osteoarthritis	68.5	39.2	6.0	Triathlon CR	Resurfacing/ No resurfacing	74
Vukadin, 2017 [36]	Serbia	NR	Osteoarthrosis/Valgus deformity	67.4	45.0	2.0	Zimmer Nexgen LPS-type	Resurfacing/ No resurfacing	60
Dong, 2018 [47]	China	2013–2015	Late-stage osteoarthritis	67.7	43.0	3.0	Posterior cruciate stabilizing total knee prostheses	Resurfacing/ No resurfacing	106
Jia, 2018 [43]	China	2013–2015	Bilateral knee osteoarthritis	57.2	80.0	2.6	NR	Resurfacing/ No resurfacing	30
Kaseb, 2018 [40]	Iran	2012–2013	Non-inflammatory arthritis	64.8	16.0	0.5	Profix™ Total Knee System	Resurfacing/ No resurfacing	50
Ha, 2019 [42]	China	2011–2017	Bilateral knee osteoarthritis	65.2	63.3	5.5	Stryker Scorpio NRG	Resurfacing/ No resurfacing	120
Chawla, 2019 [49]	India	2011–2013	Osteoarthritis (not specified)	NR	20.0	5.0	NR	Resurfacing/ No resurfacing	100
Kaseb, 2019 [41]	Iran	2014–2017	Primary osteoarthritis	66.7	20.5	0.7	Zimmer Nexgen	Resurfacing/ No resurfacing	73
Thiengwittayaporn, 2019 [48]	Thailand	NR	Osteoarthritis (not otherwise specified)	68.2	17.5	1.3	Smith & Nephew Legion PS Total Knee System	Resurfacing/ No resurfacing	84
Koh, 2019 [44]	Korea	2012–2013	Primary Osteoarthritis	70.0	NR	5.0	NR	Resurfacing/ No resurfacing	98
van Raaij, 2021 [39]	Netherlands	2012–2015	Tricompartmental osteoarthritis	69.5	38.1	2.0	AGC Total Knee System, Biomet, Warsaw, IN	Resurfacing/ No resurfacing	40
Deroche, 2022 [31]	France	2017–2018	Medial femorotibial osteoarthritis	69.3	58.1	1.5	Anatomic, AMPLITUDE®, Valence 26,000, FRANCE	Resurfacing/ No resurfacing	250

*NR* Not reported

### Patellar resurfacing vs. no resurfacing

#### Anterior knee pain

**Fig. 2:**
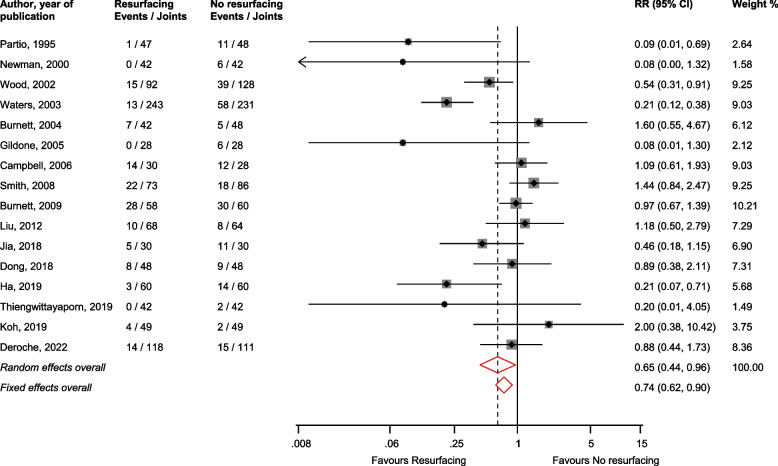
Risk of anterior knee pain comparing patellar resurfacing with no resurfacing. CI, confidence interval (bars); RR, relative risk

In pooled analysis of 16 studies [19, 31, 32, 37, 38, 42–44, 46–48, 51, 52, 55, 57, 58], patellar resurfacing reduced the risk of anterior knee pain compared with no resurfacing: RR (CI) = 0.65 (0.44–0.96; *I*^2^ = 70%; CI = 50–82%; *p* for heterogeneity < 0.01) (Fig. 2). In subgroup analyses, none of the study-level characteristics explored explained the substantial heterogeneity between studies (Supplementary Material 8).

#### Reoperations and revisions

**Fig. 3:**
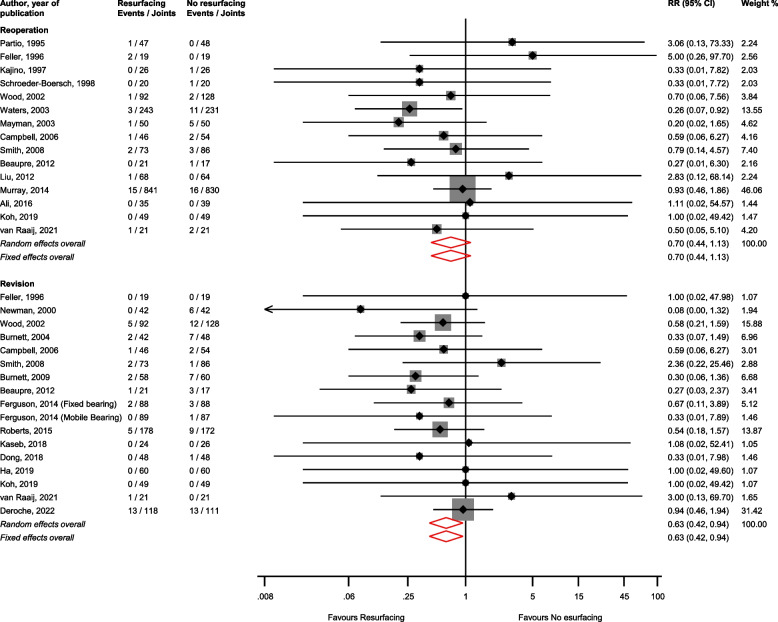
Risk of reoperation and revision comparing patellar resurfacing with no resurfacing. CI, confidence interval (bars); RR, relative risk

There was no significant difference in the risk of reoperation (15 studies) [1, 29, 35, 37–39, 44–46, 50, 53, 55–58] comparing patellar resurfacing vs. no resurfacing: RR (CIs) = 0.70 (0.44–1.13; *I*^2^ = 0%; CI = 0–54%; *p* for heterogeneity = 0.87); however patellar resurfacing reduced the risk of revision (17 studies) [19, 31, 33, 39, 40, 42, 44, 47, 51–58]: RR (CIs) = 0.63 (0.42–0.94; *I*^2^ = 0%; CI = 0–51%; *p* for heterogeneity = 0.95) (Fig. 3).

#### Other complications

**Fig. 4:**
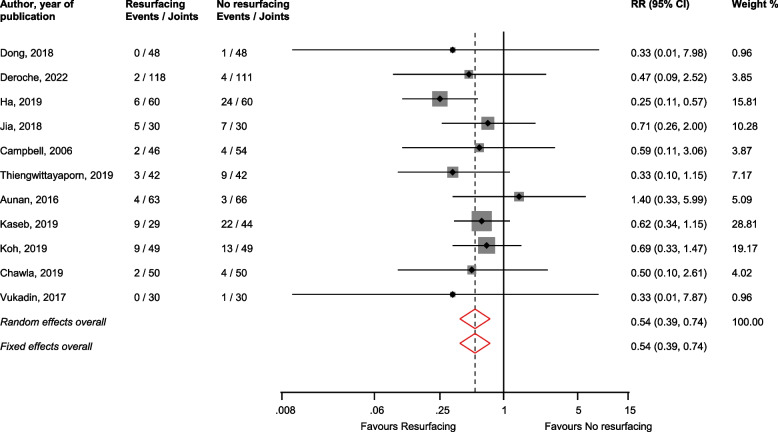
Risk of other complications comparing patellar resurfacing with no resurfacing. CI, confidence interval (bars); RR, relative risk

In pooled analysis of 11 studies [30, 31, 36, 41–44, 47–49, 55], patellar resurfacing reduced the risk of other complications (e.g. patellar dislocation, crepitus, clunk syndrome) compared with no resurfacing: RR (CIs) = 0.54 (0.39–0.74; *I*^2^ = 0%; CI = 0–60%; *p* for heterogeneity = 0.75) (Fig. 4).

#### Function

##### KSS

**Fig. 5:**
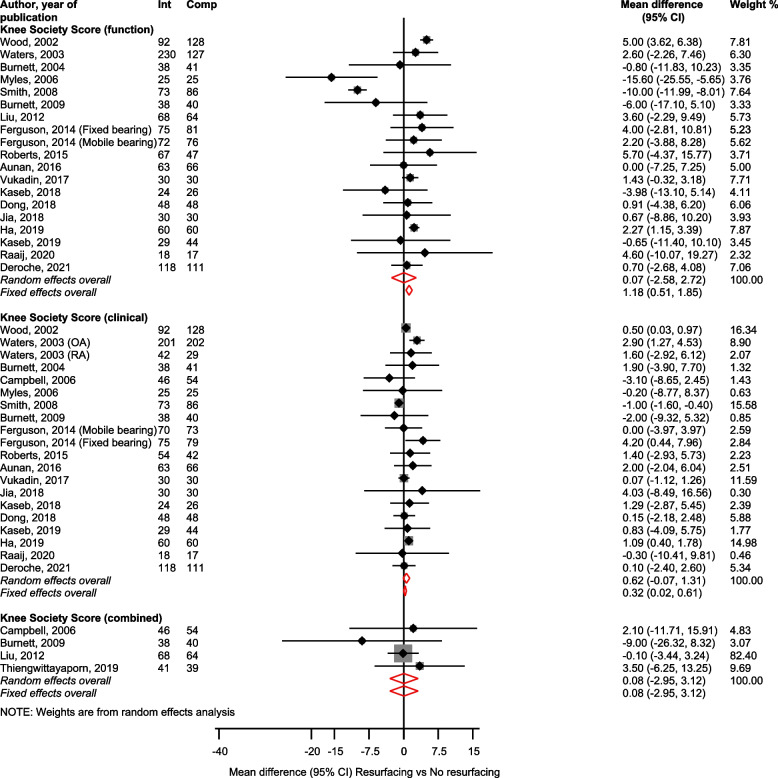
Patellar resurfacing versus no resurfacing and Knee Society Scores. CI, confidence interval (bars); Int, resurfacing; Comp, no resurfacing

KSS Function (19 studies) [30, 31, 33, 34, 36, 37, 39–43, 46, 47, 51, 52, 54, 57, 58], Clinical (20 studies) [30, 31, 33, 34, 36, 37, 39–43, 47, 51, 52, 54, 55, 57, 58], and Combined (4 studies) [46, 48, 52, 55] scores showed no statistically significant differences for patellar resurfacing vs. no resurfacing; mean differences (CIs) of 0.07 (-2.58–2.72), 0.62 (-0.07–1.31) and 0.08 (-2.95–3.12), respectively (Fig. 5).

##### KOOS

There were no statistically significant differences in KOOS subscales for symptoms (3 studies) [29, 30, 39], activities of daily living (ADL) (3 studies) [29, 30, 39], and Sport/recreation (3 studies) [29, 30, 39] between patellar resurfacing and no resurfacing: mean differences (CIs) of 1.72 (-1.84–5.27), 0.60 (-5.13–6.33) and 0.27 (-12.25–12.80) respectively (Supplementary Material 9). Results from a single report [41] showed no significant difference in KOOS combined score between the two intervention groups (Supplementary Material 9).

##### ROM

In pooled analysis of 7 studies [31, 33, 48, 51, 52, 54], there was no statistically significant difference in ROM comparing patellar resurfacing with no resurfacing: mean difference (CI) = -0.22 (-1.84–1.39) (Supplementary Material 10).

##### Other measures of function

There were no statistically significant differences between patellar resurfacing and no resurfacing for other measures of function such as patellar score (2 studies) [48, 56], Oxford Knee Score (6 studies) [1, 30, 33, 36, 48], and Feller’s Patellar Score (3 studies) [42, 44, 47]: mean differences (CIs) of 0.26 (-3.56–3.04), 1.09 (-0.30–2.48) and 0.66 (-1.13–2.45), respectively (Supplementary Material 11). Results from single reports [34, 39, 40, 56] showed no statistically significant differences in HSS Knee Score; WOMAC physical function, stiffness and combined scores; and Baldini score between the two intervention groups (Supplementary Material 11).

#### Pain

There were no statistically significant differences between patellar resurfacing and no resurfacing for measures of pain such as KSS pain score (2 studies) [46, 51], Visual Analogue Scale (VAS) pain (4 studies) [29, 30, 34, 36], and KOOS pain (3 studies) [29, 30, 39]: mean differences (CIs) of 1.03 (-1.14–3.21), -0.20 (-0.45–0.06) and 0.96 (-5.72–7.64), respectively (Supplementary Material 12). Results from single reports [34, 40, 57] showed no significant differences in WOMAC pain, Knee Pain Scale, VAS Pain Score and Anterior Knee Pain Score (Supplementary Material 12).

#### Health status, satisfaction and quality of life

Measures of health status and quality of life were mostly based on single reports [1, 29, 30, 33, 39, 40], which showed no statistically significant differences between patellar resurfacing and no resurfacing (Supplementary Material 13). In pooled analysis of 10 studies that reported the number of patients satisfied with their procedure [29, 37, 42, 44, 46, 47, 50–52, 57], there was no significant difference between patellar resurfacing and no resurfacing: RR (CIs) = 1.00 (0.94–1.08; *I*^2^ = 57%; CI = 14–79%; *p* for heterogeneity = 0.01) (Supplementary Material 14).

#### Publication bias

Funnel plots for risk of anterior knee pain, reoperation, revision, other complications and overall satisfaction comparing patellar resurfacing with no resurfacing which involved 10 or more studies were symmetrical on visual inspection, implying little evidence of small study effects or publication bias (Supplementary Material 15). These were consistent with Egger’s regression tests (*p-*values of 0.16, 0.94, 0.57, 0.81 and 0.24, respectively).

#### Selective resurfacing

Only one RCT evaluated outcomes of selective resurfacing, which was compared with patellar resurfacing and no resurfacing [19]. At five-year follow-up, the no resurfacing group required more reoperations (14%) than the patellar resurfacing (0%) and selective resurfacing (2%) groups. These findings were statistically significant. Using the mean Bristol Knee Score, there were little differences between the three groups.

In observational studies, there was no statistically significant difference in the risk of complications (2 studies) [20, 59] comparing selective resurfacing with no resurfacing: RR (CIs) = 1.06 (0.92–1.22). Selective resurfacing reduced the risk of revision (4 studies) [20, 21, 59, 61] and increased the risk of pain (2 studies) [20, 60] compared with no resurfacing: RRs (CIs) of 0.92 (0.85–0.99) and 1.25 (1.04–1.50), respectively (Supplementary Material 16).

In pooled analysis of two observational studies [20, 21], selective resurfacing was associated with an increased risk of revision compared with patellar resurfacing: RR (CIs) = 1.14 (1.05–1.22) (Supplementary Material 17). Results from one report [20] showed an increased risk of complications and pain with selective resurfacing over patellar resurfacing (Supplementary Material 17).

#### Measures of function and pain

Improved HSS Knee score (2 studies) [60, 61] and function score (2 studies) [60, 61] were seen with selective resurfacing compared with no resurfacing, with a decrease in the ROM (2 studies) [60, 61]: mean differences (CIs) of 3.47 (2.29–4.65), 1.99 (0.78–3.19) and -2.57 (-4.67,-0.47), respectively (Supplementary Material 18). Results from single reports [20, 60] showed no significant difference in pain score, but a significant improvement in the Oxford Knee Score comparing selective resurfacing vs. no resurfacing (Supplementary Material 18).

### GRADE summary of findings

The GRADE working group recommends up to 7 patient-important outcomes to be listed in the “summary of findings” tables in systematic reviews. Given that all the outcomes assessed were important, we selected the outcomes based on their frequency of reporting. GRADE ratings for the outcomes of anterior knee pain, reoperation, revision, KSS Function, KSS Clinical, ROM and overall satisfaction comparing patellar resurfacing vs. no resurfacing are reported in Supplementary Material 19. GRADE quality of the evidence ranged from high to very low.

## Discussion

Whether or not to perform patellar resurfacing during primary TKR is controversial, with advantages and disadvantages recognised for each approach [15]. Our systematic review has demonstrated that many trials exist comparing resurfacing with no resurfacing (33 RCTs involving 5,499 primary TKRs). These show that patellar resurfacing is associated with a reduced risk of anterior knee pain, revision surgery, and complications, although PROMs are similar between both strategies. The quality of the evidence ranged from high to very low. Level 1 evidence for selective resurfacing is lacking with only one small RCT available from over 20 years ago, but selective resurfacing is the most common strategy used by UK surgeons and in many other countries [12, 14, 20, 21]. Findings from the limited observational data were mostly inconsistent. Selective resurfacing decreased revision rates when compared with no resurfacing, although revision rates for selective resurfacing were increased when compared with patellar resurfacing. Selective resurfacing increased the risk of pain when compared with each of the other resurfacing strategies.

Although there is no clinical benefit in terms of PROMs between patellar resurfacing and no resurfacing, the higher risk of revision with no resurfacing is concerning, especially as outcomes after revision are much less favourable than primary TKR [62, 63]. Many revisions are performed for anterior knee pain, which is more prevalent in those TKRs not resurfaced initially [16, 17]. A recent NJR study of 842,072 primary TKRs highlighted the scale of the problem, with a significantly increased risk of all-cause revision at ten-years with no resurfacing compared with resurfacing; this equated to 2,842 excess revision procedures compared with if all TKRs underwent resurfacing initially [62]. The largest RCT identified (*n*= 1,715) recruited from 34 UK centres with 10 years follow-up and showed no significant differences in PROMs between groups [1]. However, patellar resurfacing was estimated to be very probably cost-effective. Always resurfacing resulted in more QALYs and was cheaper overall, as the incremental costs of resurfacing during the initial TKR admission were outweighed by the costs of complications and further surgery associated with needing to resurface some of the no resurfacing group in the future. Therefore NICE currently recommend patellar resurfacing over no resurfacing [18]. Previous trials and recommendations [1, 3, 18] are gradually changing clinical practice in the UK with 43% of TKRs undergoing patellar resurfacing in 2019 compared to 36% in 2011 [64].

A recent survey of UK knee surgeons identified selective patellar resurfacing as the most common strategy for primary TKR [12]. Deciding whether or not to resurface the patella is multifactorial, with surgeons stating the twelve commonest reasons for this being the condition of the patella articular cartilage (61%), presence of inflammatory arthritis (53%), native patella thickness (49%), preoperative anterior knee pain (47%), the risk of future secondary patellar resurfacing (43%), how the native patella moves/tracks during surgery (40%), the risk of postoperative anterior knee pain (38%), patient age (26%), the risk of patella fracture/extensor mechanism failure postoperatively (23%), level of TKR constraint used (22%), brand of TKR (20%), and the risk of needing revision secondary to patellar component loosening/failure (11%) [12]. In addition to the above indications, other studies have also cited indications for selective patellar resurfacing to include patient gender, weight and patella anatomy [13]. However our work demonstrated that little evidence is available to support selective patellar resurfacing. Only one small RCT from 20 years ago exists, which randomised 125 patients to resurfacing, no resurfacing or selective resurfacing [19]. The no resurfacing group required more reoperations at five-years compared with the other two groups [19]. NICE could not make any recommendations about selective resurfacing as there was only one RCT available [19]. However our updated review confirmed none of the five RCTs published since the NICE guidelines included a selective resurfacing arm. It was therefore important to consider the observational evidence available, given this is the only data that can help inform clinical decision-making, although it is recognised that such evidence is weaker compared to RCTs. The five observational studies we included suggested that currently selective resurfacing appears to have little clinical benefit over the other resurfacing strategies used. This is an important clinical concern as aside from the UK, selective patellar resurfacing is the most commonly used approach for primary TKR in many other countries worldwide including Australia, New Zealand and Denmark [14, 20, 21]. Therefore many TKR patients worldwide may be at unnecessary risk by receiving selective patellar resurfacing.

As life expectancy increases, the growing burden of osteoarthritis is expected to proportionally increase and there will continue to be increasing demand for primary TKR. This will be compounded by substantial delays in elective operating for the last two-years due to COVID-19. Many patients have suboptimal outcomes following TKR and their continued pain and need for further surgery may relate to the choices made about their patella during TKR surgery. Our findings confirm that there is plenty of level 1 evidence supporting patella resurfacing over no resurfacing. We suggest that no further trials are needed comparing these two treatment options. However, there is a lack of interventional evidence available for selective resurfacing. The current observational data shows selective resurfacing has an increased revision risk compared with resurfacing, and a higher risk of pain compared with no resurfacing. This is clinically concerning as presently there is a lack of evidence to suggest that selective resurfacing has clinical benefits over other strategies, and it is possible that selective resurfacing may actually lead to harm.

### Strengths and limitations

Based on evidence from 33 RCTs and 5 observational cohort studies, our review represents an up-to-date comprehensive meta-analysis evaluating the effectiveness of all three resurfacing options in primary TKR. Detailed assessments of methodological quality of observational studies and the risk of bias of RCTs were conducted using robust validated tools. We also evaluated a comprehensive list of outcomes. Though the limitations were mostly inherent to the studies, they include: (i) a significant degree of heterogeneity among some pooled comparisons, (ii) risk estimates for the longest follow-up of each study were pooled because of the varied follow-up periods across studies; however, where there was relevant and adequate data available, we conducted subgroup analysis by the follow-up duration, and (iii) findings on selective resurfacing were largely based on observational cohorts which are methodological weaker (as there was only one small old RCT available which included patients having selective resurfacing), with those observational cohorts limited by residual confounding, regression dilution bias, reverse causation, and inability to establish causation.

## Conclusions

Our systematic review has demonstrated level 1 evidence supports primary TKR with patellar resurfacing over no resurfacing, as resurfacing is associated with a reduced risk of anterior knee pain, revision surgery, and complications; however, PROMs are similar between resurfacing and no resurfacing groups. Although selective resurfacing is the most common strategy currently used by UK surgeons and in many other countries, there is very little published research evidence available to support this approach. We recommend large high-quality RCTs involving selective patellar resurfacing and always resurfacing to establish the role of selective resurfacing, as limited observational data suggests selective resurfacing may not have clinical benefits over other strategies.

## Supplementary Information

**Additional file 1: Supplementary Material 1: **PRISMA checklist.** Supplementary Material 2.** MOOSE checklist.** Supplementary Material 3.** Literature search strategy.** Supplementary Material 4.** Reference list of studies.** Supplementary Material 5.** Risk of bias assessment for randomised controlled trials. **Supplementary Material 6.** Baseline characteristics of observational studies. **Supplementary Material 7.** NOS scores for observational studies. **Supplementary Material 8.** Risk of anterior knee pain comparing patellar resurfacing with no resurfacing, by study-level characteristics. **Supplementary Material 9.** Patellar resurfacing versus no resurfacing and KOOS scale. **Supplementary Material 10.** Patellar resurfacing versus no resurfacing and range of movement. **Supplementary Material 11.** Patellar resurfacing versus no resurfacing and other measures of function. **Supplementary Material 12.** Patellar resurfacing versus no resurfacing and measures of pain. **Supplementary Material 13.** Patellar resurfacing versus no resurfacing and measures of health status, satisfaction, and quality of life. **Supplementary Material 14.** Patellar resurfacing versus no resurfacing and overall satisfaction. **Supplementary Material 15.** Funnel plots for risk of anterior knee pain, reoperations and revisions. **Supplementary Material 16.** Risk of revision, complications and pain comparing selective resurfacing with no resurfacing in observational cohort studies. **Supplementary Material 17.** Risk of revision, complications and pain comparing selective resurfacing with resurfacing in observational cohort studies. **Supplementary Material 18.** Selective resurfacing versus non-resurfacing and measures of function and pain in observational cohort studies. **Supplementary Material 19.** GRADE summary of findings.

## Data Availability

The datasets generated and/or analysed during the current study are not publicly available in a dedicated repository because the data extracted and analysed for this study is freely available by accessing the individual articles referenced in this publication. However the datasets may be available from the corresponding author on reasonable request.

## Abbreviations

CI: Confidence intervals
OKS: Oxford Knee Score
PROMS: Patient reported outcome measures
RCT: Randomised-control trial
RR: Relative risk
TKR: Total knee replacement
