# Supplementary material for: Clinical effectiveness of patellar resurfacing, no resurfacing and selective resurfacing in primary total knee replacement: systematic review and meta-analysis of interventional and observational evidence
Source: BMC Musculoskelet Disord. 2022 Oct 22;23:932. doi: 10.1186/s12891-022-05877-7 (PMC9587662; doi:10.1186/s12891-022-05877-7)
Supplement: Supplementary file 1 — Additional file 1: Supplementary Material 1: PRISMA checklist. Supplementary Material 2. MOOSE checklist. Supplementary Material 3. Literature search strategy. Supplementary Material 4. Reference list of studies. Supplementary Material 5. Risk of bias assessment for randomised controlled trials. Supplementary Material 6. Baseline characteristics of observational studies. Supplementary Material 7. NOS scores for observational studies. Supplementary Material 8. Risk of anterior knee pain comparing patellar resurfacing with no resurfacing, by study-level characteristics. Supplementary Material 9. Patellar resurfacing versus no resurfacing and KOOS scale. Supplementary Material 10. Patellar resurfacing versus no resurfacing and range of movement. Supplementary Material 11. Patellar resurfacing versus no resurfacing and other measures of function. Supplementary Material 12. Patellar resurfacing versus no resurfacing and measures of pain. Supplementary Material 13. Patellar resurfacing versus no resurfacing and measures of health status, satisfaction, and quality of life. Supplementary Material 14. Patellar resurfacing versus no resurfacing and overall satisfaction. Supplementary Material 15. Funnel plots for risk of anterior knee pain, reoperations and revisions. Supplementary Material 16. Risk of revision, complications and pain comparing selective resurfacing with no resurfacing in observational cohort studies. Supplementary Material 17. Risk of revision, complications and pain comparing selective resurfacing with resurfacing in observational cohort studies. Supplementary Material 18. Selective resurfacing versus non-resurfacing and measures of function and pain in observational cohort studies. Supplementary Material 19. GRADE summary of findings. [file 12891_2022_5877_MOESM1_ESM.docx]

**Supplementary Material**

| **Supplementary Material 1** | PRISMA checklist |
| --- | --- |
| **Supplementary Material 2** | MOOSE checklist |
| **Supplementary Material 3** | Literature search strategy |
| **Supplementary Material 4** | Reference list of included studies |
| **Supplementary Material 5** | Risk of bias assessment |
| **Supplementary Material 6** | Baseline characteristics of observational studies |
| **Supplementary Material 7** | NOS scores for observational studies |
| **Supplementary Material 8** | Risk of anterior knee pain comparing patellar resurfacing with no resurfacing, by study-level characteristics |
| **Supplementary Material 9** | Patellar resurfacing versus no resurfacing and KOOS scale |
| **Supplementary Material 10** | Patellar resurfacing versus no resurfacing and range of movement |
| **Supplementary Material 11** | Patellar resurfacing versus no resurfacing and other measures of function |
| **Supplementary Material 12** | Patellar resurfacing versus no resurfacing and measures of pain |
| **Supplementary Material 13** | Patellar resurfacing versus no resurfacing and measures of health status, satisfaction and quality of life |
| **Supplementary Material 14** | Patellar resurfacing versus no resurfacing and overall satisfaction |
| **Supplementary Material 15** | Funnel plots for risk of anterior knee pain, reoperations, and revisions |
| **Supplementary Material 16** | Risk of revision, complications and pain comparing selective resurfacing with no resurfacing in observational cohort studies |
| **Supplementary Material 17** | Risk of revision, complications and pain comparing selective resurfacing with resurfacing in observational cohort studies |
| **Supplementary Material 18** | Selective resurfacing versus no resurfacing and measures of function and pain in observational cohort studies |
| **Supplementary Material 19** | GRADE summary of findings |

**Supplementary Material 1.** PRISMA checklist

| **Section/topic** | **Item No** | **Checklist item** | **Reported on page No** |
| --- | --- | --- | --- |
| **Title** | | | |
| Title | 1 | Identify the report as a systematic review, meta-analysis, or both | 1 |
| **Abstract** | | | |
| Structured summary | 2 | Provide a structured summary including, as applicable, background, objectives, data sources, study eligibility criteria, participants, interventions, study appraisal and synthesis methods, results, limitations, conclusions and implications of key findings, systematic review registration number | 2 |
| **Introduction** | | | |
| Rationale | 3 | Describe the rationale for the review in the context of what is already known | Introduction |
| Objectives | 4 | Provide an explicit statement of questions being addressed with reference to participants, interventions, comparisons, outcomes, and study design (PICOS) | Introduction |
| **Methods** | | | |
| Protocol and registration | 5 | Indicate if a review protocol exists, if and where it can be accessed (such as web address), and, if available, provide registration information including registration number | Methods |
| Eligibility criteria | 6 | Specify study characteristics (such as PICOS, length of follow-up) and report characteristics (such as years considered, language, publication status) used as criteria for eligibility, giving rationale | Methods |
| Information sources | 7 | Describe all information sources (such as databases with dates of coverage, contact with study authors to identify additional studies) in the search and date last searched | Methods |
| Search | 8 | Present full electronic search strategy for at least one database, including any limits used, such that it could be repeated | Supplementary Material 3 |
| Study selection | 9 | State the process for selecting studies (that is, screening, eligibility, included in systematic review, and, if applicable, included in the meta-analysis) | Methods |
| Data collection process | 10 | Describe method of data extraction from reports (such as piloted forms, independently, in duplicate) and any processes for obtaining and confirming data from investigators | Methods |
| Data items | 11 | List and define all variables for which data were sought (such as PICOS, funding sources) and any assumptions and simplifications made | Methods |
| Risk of bias in individual studies | 12 | Describe methods used for assessing risk of bias of individual studies (including specification of whether this was done at the study or outcome level), and how this information is to be used in any data synthesis | Methods |
| Summary measures | 13 | State the principal summary measures (such as risk ratio, difference in means). | Methods |
| Synthesis of results | 14 | Describe the methods of handling data and combining results of studies, if done, including measures of consistency (such as I^2^ statistic) for each meta-analysis | Methods |
| Risk of bias across studies | 15 | Specify any assessment of risk of bias that may affect the cumulative evidence (such as publication bias, selective reporting within studies) | Methods |
| Additional analyses | 16 | Describe methods of additional analyses (such as sensitivity or subgroup analyses, meta-regression), if done, indicating which were pre-specified | Methods |
| **Results** | | | |
| Study selection | 17 | Give numbers of studies screened, assessed for eligibility, and included in the review, with reasons for exclusions at each stage, ideally with a flow diagram | Results and Figure 1 |
| Study characteristics | 18 | For each study, present characteristics for which data were extracted (such as study size, PICOS, follow-up period) and provide the citations | Results; Table 1; SM 5 |
| Risk of bias within studies | 19 | Present data on risk of bias of each study and, if available, any outcome-level assessment (see item 12). | Results; Table 1; |
| Results of individual studies | 20 | For all outcomes considered (benefits or harms), present for each study (a) simple summary data for each intervention group and (b) effect estimates and confidence intervals, ideally with a forest plot | Results; Figures 2-5; SM 7-18 |
| Synthesis of results | 21 | Present results of each meta-analysis done, including confidence intervals and measures of consistency | Results; Figures 2-5; SM 7-18 |
| Risk of bias across studies | 22 | Present results of any assessment of risk of bias across studies (see item 15) | SM 4 and 6 |
| Additional analysis | 23 | Give results of additional analyses, if done (such as sensitivity or subgroup analyses, meta-regression) (see item 16) | SM 7 |
| **Discussion** | | | |
| Summary of evidence | 24 | Summarise the main findings including the strength of evidence for each main outcome; consider their relevance to key groups (such as health care providers, users, and policy makers) | Discussion |
| Limitations | 25 | Discuss limitations at study and outcome level (such as risk of bias), and at review level (such as incomplete retrieval of identified research, reporting bias) | Discussion |
| Conclusions | 26 | Provide a general interpretation of the results in the context of other evidence, and implications for future research | Discussion |
| **Funding** | | | |
| Funding | 27 | Describe sources of funding for the systematic review and other support (such as supply of data) and role of funders for the systematic review | After discussion |

**Supplementary Material 2.** MOOSE checklist

**Clinical effectiveness of patella resurfacing, selective resurfacing and no resurfacing in primary total knee replacement: systematic review and meta-analysis of interventional and observational evidence**

| **Criteria** | | **Brief description of how the criteria were handled in the review** |
| --- | --- | --- |
| **Reporting of background** | |  |
| √ | Problem definition | The effectiveness of patella resurfacing, selective resurfacing, and no resurfacing in primary total knee replacement (TKR) is unclear. |
| √ | Hypothesis statement | There are no differences in the effectiveness of patella resurfacing, selective resurfacing, and no resurfacing in TKR |
| √ | Description of study outcomes | Patient reported outcome measures (PROMs), complications, and further surgery |
| √ | Type of exposure | Patella resurfacing, selective resurfacing, and no resurfacing |
| √ | Type of study designs used | Randomised controlled trials (RCTs) and observational cohorts |
| √ | Study population | Primary TKR |
| **Reporting of search strategy should include** | |  |
| √ | Qualifications of searchers | Setor K. Kunutsor, PhD |
| √ | Search strategy, including time period included in the synthesis and keywords | Time period: from inception to November 2021  The detailed search strategy can be found in Supplementary Material 3 |
| √ | Databases and registries searched | MEDLINE, EMBASE, Web of Science, and Cochrane databases |
| √ | Search software used, name and version, including special features | OvidSP was used to search EMBASE and MEDLINE  EndNote used to manage references |
| √ | Use of hand searching | We searched bibliographies of retrieved papers |
| √ | List of citations located and those excluded, including justifications | Details of the literature search process are outlined in the flow chart. The citation list for excluded studies are available on request. |
| √ | Method of addressing articles published in languages other than English | Not applicable |
| √ | Method of handling abstracts and unpublished studies | Abstracts with no full text publications were not included. |
| √ | Description of any contact with authors | None |
| **Reporting of methods should include** | |  |
| √ | Description of relevance or appropriateness of studies assembled for assessing the hypothesis to be tested | Detailed inclusion and exclusion criteria are described in the Methods section. |
| √ | Rationale for the selection and coding of data | Data extracted from each of the studies were relevant to the population characteristics, study design, exposure, and outcome. |
| √ | Assessment of confounding | Not applicable |
| √ | Assessment of study quality, including blinding of quality assessors; stratification or regression on possible predictors of study results | Risk of bias in RCTs was assessed using the Cochrane Risk of Bias Tool  Methodological quality of observational cohort studies was assessed using the nine-star Newcastle-Ottawa Scale. |
| √ | Assessment of heterogeneity | Heterogeneity of the studies was quantified with I^2^ statistic that provides the relative amount of variance of the summary effect due to the between-study heterogeneity |
| √ | Description of statistical methods in sufficient detail to be replicated | Description of methods of meta-analyses. We performed random effects meta-analysis with Stata 16. |
| √ | Provision of appropriate tables and graphics | Table 1; Figures 1-5; Supplementary Materials 4-18 |
| **Reporting of results should include** | |  |
| √ | Graph summarizing individual study estimates and overall estimate | Figures 2-5; Supplementary Materials 7-18 |
| √ | Table giving descriptive information for each study included | Table 1; Supplementary Material 5 |
| √ | Results of sensitivity testing | Not applicable |
| √ | Indication of statistical uncertainty of findings | 95% confidence intervals were presented with all summary estimates, I^2^ values and results of sensitivity analyses |
| **Reporting of discussion should include** | |  |
| √ | Quantitative assessment of bias | Risk of bias assessment discussed. GRADE quality of evidence reported. |
| √ | Justification for exclusion | All studies were excluded based on the pre-defined inclusion criteria in methods section. |
| √ | Assessment of quality of included studies | Brief discussion included in ‘Methods’ section |
| **Reporting of conclusions should include** | |  |
| √ | Consideration of alternative explanations for observed results | Discussion |
| √ | Generalization of the conclusions | Discussed in the context of the results. |
| √ | Guidelines for future research | Large-scale definitive trials warranted, especially for selective resurfacing. |
| √ | Disclosure of funding source | In “Acknowledgement” section |

**Supplementary Material 3.** Literature search strategy

Relevant studies, published from inception to 06 November 2021 (date last searched), were identified through electronic searches using MEDLINE, EMBASE, and Cochrane databases. Electronic searches were supplemented by scanning reference lists of articles identified for all relevant studies (including review articles) and by hand searching of relevant journals.

| Ovid MEDLINE 1946-Present  1 exp Arthroplasty, Replacement, Knee/ (23752)  2 exp Knee Joint/ (60288)  3 exp Knee Prosthesis/ (11648)  4 TKR.mp. (2034)  5 TKA.mp. (10724)  6 exp Patella/ (9879)  7 patell$.mp. (25707)  8 patella resurfacing.mp. (140)  9 resurf$.mp. (7037)  10 re-surf$.mp. (67)  11 ("clinical trial" or "clinical trial, phase i" or "clinical trial, phase ii" or clinical trial, phase iii or clinical trial, phase iv or controlled clinical trial or "multicenter study" or "randomized controlled trial").pt. or double-blind method/ or clinical trials as topic/ or clinical trials, phase i as topic/ or clinical trials, phase ii as topic/ or clinical trials, phase iii as topic/ or clinical trials, phase iv as topic/ or controlled clinical trials as topic/ or randomized controlled trials as topic/ or early termination of clinical trials as topic/ or multicenter studies as topic/ or ((randomi?ed adj7 trial*) or (controlled adj3 trial*) or (clinical adj2 trial*) or ((single or doubl* or tripl* or treb*) and (blind* or mask*))).ti,ab,kw. or ("4 arm" or "four arm").ti,ab,kw. (1622619)  12 cohort studies/ or longitudinal studies/ or follow-up studies/ or prospective studies/ or retrospective studies/ or cohort.ti,ab. or longitudinal.ti,ab. or prospective.ti,ab. or retrospective.ti,ab. (2605853)  13 1 or 2 or 3 or 4 or 5 (82894)  14 6 or 7 or 8 or 9 or 10 (32006)  15 11 or 12 (3782576)  16 13 and 14 and 15 (3967)  17 limit 16 to humans (3890)  Each part was specifically translated for searching the other databases (EMBASE and Cochrane databases) |
| --- |

**Supplementary Material 4.** Reference list of studies

1. Ali A, Lindstrand A, Nilsdotter A, Sundberg M. Similar patient-reported outcomes and performance after total knee arthroplasty with or without patellar resurfacing. Acta Orthop 2016;**87**(3):274-9.

2. Aunan E, Naess G, Clarke-Jenssen J, Sandvik L, Kibsgard TJ. Patellar resurfacing in total knee arthroplasty: functional outcome differs with different outcome scores: A randomized, double-blind study of 129 knees with 3 years of follow-up. Acta Orthop 2016;**87**(2):158-64.

3. Beaupre L, Secretan C, Johnston DW, Lavoie G. A randomized controlled trial comparing patellar retention versus patellar resurfacing in primary total knee arthroplasty: 5-10 year follow-up. BMC Res Notes 2012;**5**:273.

4. Burnett RS, Boone JL, Rosenzweig SD, Steger-May K, Barrack RL. Patellar resurfacing compared with nonresurfacing in total knee arthroplasty. A concise follow-up of a randomized trial. J Bone Joint Surg Am 2009;**91**(11):2562-7.

5. Burnett RS, Haydon CM, Rorabeck CH, Bourne RB. Patella resurfacing versus nonresurfacing in total knee arthroplasty: results of a randomized controlled clinical trial at a minimum of 10 years' followup. Clin Orthop Relat Res 2004(428):12-25.

6. Campbell DG, Duncan WW, Ashworth M, Mintz A, Stirling J, Wakefield L, Stevenson TM. Patellar resurfacing in total knee replacement: a ten-year randomised prospective trial. J Bone Joint Surg Br 2006;**88**(6):734-9.

7. Chawla L, Bandekar SM, Dixit V, P A, Krishnamoorthi A, Mummigatti S. Functional outcome of patellar resurfacing vs non resurfacing in Total Knee Arthoplasty in elderly: A prospective five year follow-up study. Journal of Arthroscopy and Joint Surgery 2019;**6**(1):65-69.

8. Deroche E, Batailler C, Swan J, Sappey-Marinier E, Neyret P, Servien E, Lustig S. No difference between resurfaced and non-resurfaced patellae with a modern prosthesis design: a prospective randomized study of 250 total knee arthroplasties. Knee Surg Sports Traumatol Arthrosc 2021.

9. Dong Y, Li T, Zheng Z, Xiang S, Weng X. Adding Patella Resurfacing After Circumpatellar Electrocautery Did Not Improve the Clinical Outcome in Bilateral Total Knee Arthroplasty in Chinese Population: A Prospective Randomized Study. J Arthroplasty 2018;**33**(4):1057-1061.

10. Feller JA, Bartlett RJ, Lang DM. Patellar resurfacing versus retention in total knee arthroplasty. J Bone Joint Surg Br 1996;**78**(2):226-8.

11. Ferguson KB, Bailey O, Anthony I, James PJ, Stother IG, M JGB. A prospective randomised study comparing rotating platform and fixed bearing total knee arthroplasty in a cruciate substituting design--outcomes at two year follow-up. Knee 2014;**21**(1):151-5.

12. Gildone A, Manfredini M, Biscione R, Faccini R. Patella resurfacing in posterior stabilised total knee arthroplasty: a follow-up study in 56 patients. Acta Orthop Belg 2005;**71**(4):445-51.

13. Ha C, Wang B, Li W, Sun K, Wang D, Li Q. Resurfacing versus not-resurfacing the patella in one-stage bilateral total knee arthroplasty: a prospective randomized clinical trial. Int Orthop 2019;**43**(11):2519-2527.

14. Jia C, Ni M, Fu J, Li X, Li X, Chai W, Chen J. [A comparative study on effectiveness of patellar resurfacing against non-resurfacing in total knee arthroplasty]. Zhongguo Xiu Fu Chong Jian Wai Ke Za Zhi 2018;**32**(4):394-399.

15. Kajino A, Yoshino S, Kameyama S, Kohda M, Nagashima S. Comparison of the results of bilateral total knee arthroplasty with and without patellar replacement for rheumatoid arthritis. A follow-up note. J Bone Joint Surg Am 1997;**79**(4):570-4.

16. Kaseb MH, Mortazavi J, Ayati Firoozabadi M, Toofan H. Comparison between Patellar Resurfacing and Retention in Total Knee Arthroplasty Regarding the Postoperative Satisfaction of Patients and Patellar Crepitus. Arch Bone Jt Surg 2019;**7**(5):441-444.

17. Kaseb MH, Tahmasebi MN, Mortazavi SJ, Sobhan MR, Nabian MH. Comparison of Clinical Results between Patellar Resurfacing and Non-resurfacing in Total Knee Arthroplasty: A Short Term Evaluation. Arch Bone Jt Surg 2018;**6**(2):124-129.

18. Koh IJ, Kim MS, Sohn S, Song KY, Choi NY, In Y. Patients undergoing total knee arthroplasty using a contemporary patella-friendly implant are unaware of any differences due to patellar resurfacing. Knee Surg Sports Traumatol Arthrosc 2019;**27**(4):1156-1164.

19. Liu ZT, Fu PL, Wu HS, Zhu Y. Patellar reshaping versus resurfacing in total knee arthroplasty - Results of a randomized prospective trial at a minimum of 7 years' follow-up. Knee 2012;**19**(3):198-202.

20. Maney AJ, Koh CK, Frampton CM, Young SW. Usually, Selectively, or Rarely Resurfacing the Patella During Primary Total Knee Arthroplasty: Determining the Best Strategy. J Bone Joint Surg Am 2019;**101**(5):412-420.

21. Maradit-Kremers H, Haque OJ, Kremers WK, Berry DJ, Lewallen DG, Trousdale RT, Sierra RJ. Is Selectively Not Resurfacing the Patella an Acceptable Practice in Primary Total Knee Arthroplasty? J Arthroplasty 2017;**32**(4):1143-1147.

22. Mayman D, Bourne RB, Rorabeck CH, Vaz M, Kramer J. Resurfacing versus not resurfacing the patella in total knee arthroplasty: 8- to 10-year results. J Arthroplasty 2003;**18**(5):541-5.

23. Misra AN, Smith RB, Fiddian NJ. Five year results of selective patellar resurfacing in cruciate sparing total knee replacements. Knee 2003;**10**(2):199-203.

24. Murray DW, MacLennan GS, Breeman S, Dakin HA, Johnston L, Campbell MK, Gray AM, Fiddian N, Fitzpatrick R, Morris RW, Grant AM, group KAT. A randomised controlled trial of the clinical effectiveness and cost-effectiveness of different knee prostheses: the Knee Arthroplasty Trial (KAT). Health Technol Assess 2014;**18**(19):1-235, vii-viii.

25. Myles CM, Rowe PJ, Nutton RW, Burnett R. The effect of patella resurfacing in total knee arthroplasty on functional range of movement measured by flexible electrogoniometry. Clin Biomech (Bristol, Avon) 2006;**21**(7):733-9.

26. Newman JH, Ackroyd CE, Shah NA, Karachalios T. Should the patella be resurfaced during total knee replacement? The Knee 2000;**7**(1):17-23.

27. Park SJ, Jung YB, Jeong HJ, Shin HK, Jung HJ, Lim JJ, Yoon JW, Kim E. Long-term results of primary total knee arthroplasty with and without patellar resurfacing. Acta Med Okayama 2010;**64**(5):331-8.

28. Partio E. Comparison of patellar resurfacing and nonresurfacing in total knee arthroplasty : a prospective ramdamized study. J Orthop Rheumatol 1995;**8**:69-74.

29. Raaij TMV, Meij EV, Vries AJ, Raay J. Patellar Resurfacing Does Not Improve Clinical Outcome in Patients with Symptomatic Tricompartmental Knee Osteoarthritis. An RCT Study of 40 Patients Receiving Primary Cruciate Retaining Total Knee Arthroplasty. J Knee Surg 2020.

30. Roberts DW, Hayes TD, Tate CT, Lesko JP. Selective patellar resurfacing in total knee arthroplasty: a prospective, randomized, double-blind study. J Arthroplasty 2015;**30**(2):216-22.

31. Schroeder-Boersch H, Scheller G, Fischer J, Jani L. Advantages of patellar resurfacing in total knee arthroplasty. Two-year results of a prospective randomized study. Arch Orthop Trauma Surg 1998;**117**(1-2):73-8.

32. Smith AJ, Wood DJ, Li MG. Total knee replacement with and without patellar resurfacing: a prospective, randomised trial using the profix total knee system. J Bone Joint Surg Br 2008;**90**(1):43-9.

33. Thiengwittayaporn S, Srungboonmee K, Chiamtrakool B. Resurfacing in a Posterior-Stabilized Total Knee Arthroplasty Reduces Patellar Crepitus Complication: A Randomized, Controlled Trial. J Arthroplasty 2019;**34**(9):1969-1974.

34. Vertullo CJ, Graves SE, Cuthbert AR, Lewis PL. The Effect of Surgeon Preference for Selective Patellar Resurfacing on Revision Risk in Total Knee Replacement: An Instrumental Variable Analysis of 136,116 Procedures from the Australian Orthopaedic Association National Joint Replacement Registry. J Bone Joint Surg Am 2019;**101**(14):1261-1270.

35. Vukadin OB, Blagojevic ZB, Bascarevic ZL, Slavkovic NS, Stevanovic V, Vukomanovic BD. The Importance of Patellar Resurfacing in Total Knee Arthroplasty for Symptomatic Valgus Degenerative Deformity. Acta Chir Orthop Traumatol Cech 2017;**84**(1):30-34.

36. Waters TS, Bentley G. Patellar resurfacing in total knee arthroplasty. A prospective, randomized study. J Bone Joint Surg Am 2003;**85**(2):212-7.

37. Wood DJ, Smith AJ, Collopy D, White B, Brankov B, Bulsara MK. Patellar resurfacing in total knee arthroplasty: a prospective, randomized trial. J Bone Joint Surg Am 2002;**84**(2):187-93.

**Supplementary Material 5.** Risk of bias assessment for randomised controlled trials

**Supplementary Material 6.** Baseline characteristics of observational studies

| **Author, year of publication** | **Location** | **Baseline year of study** | **Population** | **Mean/median age (years)** | **% Male** | **Implant design** | **Interventions compared** | **No. of participants or joint replacements** |
| --- | --- | --- | --- | --- | --- | --- | --- | --- |
| Maney, 2019 | New Zealand | 1999-2015 | Osteoarthritis (not otherwise specified) | 67.67 | 49.5 | NexGen (Zimmer), the Triathlon (Stryker), the LCS Knee System (DePuy Synthes), and the Genesis II (Smith & Nephew) | Resurfacing/ No resurfacing/ Selective resurfacing | 32,148 |
| Maradit-Kremers, 2017 | America | 1985-2010 | NR | 68.6 | 44.1 | PFC, Nexgen Legacy, Sigma, PCA, Genesis | Selective resurfacing/ No resurfacing | 21,371 |
| Misra, 2003 | United Kingdom | 1996-2001 | Osteoarthritis and Rheumatoid Arthritis | 67.5 | 37.1 | PFC Sigma© | Selective resurfacing/ No resurfacing | 124 |
| Park, 2010 | Korea | 1990-1999 | Degenerative osteoarthritis | 64.3 | 6.6 | LCS, DePuy, Warsaw, IN, USA | Selective resurfacing/ No resurfacing | 44 |
| Vertullo, 2019 | Australia | 1999-2016 | Osteoarthritis (not otherwise specified) | 68.7 | 41.9 | NR | Resurfacing/ No resurfacing/ Selective resurfacing | 136116 |

**Supplementary Material 7.**

NOS scores for observational studies

| **Author** | **Year of publication** | **Selection** | **Comparability** | **Outcome** | **Total score (max. 9)** |
| --- | --- | --- | --- | --- | --- |
| Maney et al. | 2019 | 4 | 2 | 2 | 8 |
| Maradit-Kremer et al. | 2017 | 4 | 2 | 3 | 9 |
| Misra et al. | 2003 | 4 | 0 | 3 | 7 |
| Park et al. | 2010 | 4 | 0 | 3 | 7 |
| Vertullo et al. | 2019 | 4 | 2 | 3 | 9 |

NOS, Newcastle-Ottawa Scale

**Supplementary Material 8.** Risk of anterior knee pain comparing patellar resurfacing with no resurfacing, by study-level characteristics

**Supplementary Material 9.** Patellar resurfacing versus no resurfacing and KOOS scale

ADL, activities of daily living; CI, confidence interval (bars); KOOS, Knee Injury and Osteoarthritis Outcome Score

**Supplementary Material 10.** Patellar resurfacing versus no resurfacing and range of movement

CI, confidence interval (bars)

**Supplementary Material 11.** Patellar resurfacing versus no resurfacing and other measures of function

CI, confidence interval (bars); HSS, Hospital for Special Surgery; WOMAC, Western Ontario and McMaster Universities Osteoarthritis Index

**Supplementary Material 12.** Patellar resurfacing versus no resurfacing and measures of pain

CI, confidence interval (bars); KOOS, Knee Injury and Osteoarthritis Outcome Score; WOMAC, Western Ontario and McMaster Universities Osteoarthritis Index; VAS, Visual Analogue Scale

**Supplementary Material 13.** Patellar resurfacing versus no resurfacing and measures of health status, satisfaction, and quality of life

CI, confidence interval (bars); KOOS, Knee Injury and Osteoarthritis Outcome Score

**Supplementary Material 14.** Patellar resurfacing versus no resurfacing and overall satisfaction

**Supplementary Material 15.** Funnel plots for risk of anterior knee pain, reoperations and revisions

The dotted lines show 95% confidence intervals around the overall summary estimate calculated using a fixed effect model; *p*-values for bias calculated using Egger’s test were 16, .94, .57, .81 and .24, respectively, for risk of anterior knee pain, reoperation, revision, other complications and overall satisfaction, respectively

**Supplementary Material 16.** Risk of revision, complications and pain comparing selective resurfacing with no resurfacing in observational cohort studies

CI, confidence interval; RR, relative risk

**Supplementary Material 17.** Risk of revision, complications and pain comparing selective resurfacing with resurfacing in observational cohort studies

CI, confidence interval; RR, relative risk

**Supplementary Material 18.** Selective resurfacing versus non-resurfacing and measures of function and pain in observational cohort studies

CI, confidence interval; HSS, Hospital for Special Surgery; ROM, range of movement

**Supplementary Material 19.** GRADE summary of findings

| **Outcomes** | **№ of participants (studies) Follow-up** | **Certainty of the evidence (GRADE)** | **Relative effect (95% CI)** | **Anticipated absolute effects** | |
| --- | --- | --- | --- | --- | --- |
|  |  |  |  | **Risk with [No resurfacing]** | **Risk difference with [Patellar resurfacing]** |
| Anterior knee pain | 2173 (16 RCTs) | ⨁⨁◯◯ Low^a,b^ | **RR 0.65** (0.44 to 0.96) | 223 per 1,000 | **78 fewer per 1,000** (125 fewer to 9 fewer) |
| Reoperation | 3333 (15 RCTs) | ⨁⨁⨁◯ Moderate^a^ | **RR 0.70** (0.44 to 1.13) | 26 per 1,000 | **8 fewer per 1,000** (15 fewer to 3 more) |
| Revision | 2184 (17 RCTs) | ⨁⨁⨁⨁ High^a^ | **RR 0.63** (0.42 to 0.94) | 58 per 1,000 | **22 fewer per 1,000** (34 fewer to 3 fewer) |
| Knee Society Score (Function) | 2345 (19 RCTs) | ⨁◯◯◯ Very low^a,c^ | - | The mean knee Society Score (Function) was **0** | **0**  (0 to 0) |
| Knee Society Score (Clinical) | 2405 (20 RCTs) | ⨁⨁◯◯ Low^a,d^ | - | The mean knee Society Score (Clinical) was **0** | **0**  (0 to 0) |
| Range of movement | 830 (7 RCTs) | ⨁⨁⨁◯ Moderate^a^ | - | The mean range of movement was **0** | **0**  (0 to 0) |
| Overall Satisfaction | 1446 (10 RCTs) | ⨁⨁◯◯ Low^a,e^ | **RR 1.00** (0.94 to 1.08) | 723 per 1,000 | **0 fewer per 1,000** (43 fewer to 58 more) |
| ***The risk in the intervention group** (and its 95% confidence interval) is based on the assumed risk in the comparison group and the **relative effect** of the intervention (and its 95% CI).  **CI:** confidence interval; **RR:** risk ratio  **Explanations**  a. High risk of bias in some domains  b. I-squared value of 70%  c. I-squared value of 89.5%  d. I-squared value of 54.5%  e. I-squared value of 57% | | | | | |
| **GRADE Working Group grades of evidence** **High certainty:** we are very confident that the true effect lies close to that of the estimate of the effect. **Moderate certainty:** we are moderately confident in the effect estimate: the true effect is likely to be close to the estimate of the effect, but there is a possibility that it is substantially different. **Low certainty:** our confidence in the effect estimate is limited: the true effect may be substantially different from the estimate of the effect. **Very low certainty:** we have very little confidence in the effect estimate: the true effect is likely to be substantially different from the estimate of effect. | | | | | |
